# The Princess of Wales and Her Son

**Published:** 1891-12-19

**Authors:** 


### The Princess of Wales and Her Son.

In times of social upheaval and political scepticism, it is permissible to note the sayings and doings of Royal persons from a great many different points of view. The present Royal family, beginning with the Queen, have played a conspicuous part in the public and private affairs of this realm for half-a-century. All well-conditioned persons, whatever their political convictions, must recognise that Queen Victoria has exercised a potent influence on the fortunes of her country; and that that influence has made for purity, wholesomeness, and reasonableness, both in the public and in the private relations of the life of the nation. The Queen has been emphatically a wise as well as a kindly Sovereign. Her influence, which has been so potent in the State, has been, if possible, still more potent in the family and personal life of all her subjects, both high and low. Whilst there has always been conspicuous the presence of truly Royal dignity in the conduct of the national affairs, the absence of ostentatious pomp and tawdry magnificence has been still more conspicuous. Queen Victoria will always be dear to the hearts of plain, sensible, and virtuous people—the people which constitute the backbone of every noble state.

Following in the footsteps of the Queen, but showing still that if there had been no such footsteps, she would have pursued the same path, is Her Royal Highness the Princess of Wales, tenderly beloved at the present moment of every mother in the land. Prince George of Wales, the second son of his mother, and an honest sailor, has been lying between life and death for the past month. When the Princess returned in haste from her visit to Russia, she found her son too oppressed with fever and its accompanying mental torpor to give much heed to her presence. Gradually, as the fever lessened and the mind became more clear, this young man of Royal and ancient lineage, finding his mother constantly by his bedside, became unspeakably soothed and comforted by her presence.

The Princess had for some time been under a promise to pay a visit to the Duke and Duchess of Portland at Welbeck Abbey. But Prince George feels the presence of his mother so comforting and so helpful to his well-being, that Her Royal Highness has postponed her visit to Welbeck until he shall be quite recovered. We do not wish to make of this circumstance more than there is really in it. But it will gladden the heart of every mother, and, indeed, of every son in the kingdom, to find that in one Royal house the mother takes precedence of the Princess.

We do not like to call this an object lesson, because the mothers of England do not need an object lesson. It is rather an incident in the Royal home life of this country which every woman will take into her heart and preserve there as a hallowing and ennobling influence. Great is the power of Royalty in an ancient Empire to raise or to depress social life and private virtue. Great, too, are the corresponding responsibilities. Every loyal citizen of this country, which values its character and its honour so highly, is grateful with the utmost gratitude of his heart when persons in the highest places give him cause to revere them and to follow their example. We are all bound together, Queen, Princes, Princesses, and People, in one great community for mutual comfort and protection in a progressive civilisation. Our present political and social leaders lead in the ways of simplicity and of a noble humanity. These are the ways that, in a country like our own, make for the permanence of ancient institutions and the truest well-being of all classes. We venture to think that these sentiments which we have made bold to express at the present moment, will find a fervent echo in the heart of every man and woman in the land.

